# Sperm DNA fragmentation and assisted reproduction: an umbrella meta-analysis

**DOI:** 10.1186/s40001-025-03801-y

**Published:** 2026-01-07

**Authors:** Bangbei Wan, Yu Fu, Ning Ma, Zhi Zhou, Weiying Lu

**Affiliations:** 1 Reproductive Medical Center, Hainan Women and Children’s Medical Center, No. 75, Longkun South Road, Haikou, Hainan China; 2 Department of Urology, Haikou Affiliated Hospital of Central South University Xiangya School of Medicine, Haikou, China

**Keywords:** Sperm DNA fragmentation, Assisted reproductive technology, In vitro fertilization (IVF), Intracytoplasmic sperm injection (ICSI), Intrauterine insemination (IUI)

## Abstract

**Background:**

Male infertility accounts for nearly half of infertility cases worldwide. Conventional semen parameters provide limited information about sperm function. Sperm DNA fragmentation (SDF) has emerged as a potential biomarker of sperm nuclear quality, but its prognostic role in assisted reproductive technology (ART) outcomes remains controversial.

**Objectives:**

To conduct an umbrella meta-analysis of published meta-analyses assessing the association between SDF and major ART outcomes, including clinical pregnancy (CP), pregnancy loss (PL), and live birth rate (LBR).

**Methods:**

We systematically searched PubMed, Embase, Web of Science, Scopus, and the Cochrane Library through July 2025. Eligible studies were systematic reviews with meta-analyses evaluating SDF in in vitro fertilization (IVF), intracytoplasmic sperm injection (ICSI), and intrauterine insemination (IUI). Pooled risk ratios (RRs) were extracted or converted to logRRs. Evidence quality was assessed using A Measurement Tool to Assess Systematic Reviews 2 (AMSTAR-2) and the Grading of Recommendations, Assessment, Development, and Evaluation (GRADE) frameworks.

**Results:**

Eight meta-analyses comprising more than 25,000 ART cycles were included. Elevated SDF was significantly associated with reduced CP in IVF (RR = 0.662, 95% CI: 0.547–0.801) and IUI (RR = 0.467, 95% CI: 0.242–0.900), while only a weak, borderline association was observed in ICSI (RR = 0.886, 95% CI: 0.764–0.985). High SDF was strongly associated with increased PL in ICSI (RR = 2.286, 95% CI: 1.383–3.779). Evidence for LBR was limited and inconclusive. Across outcomes, certainty of evidence was graded as low due to heterogeneity, imprecision, and methodological limitations.

**Conclusions:**

Elevated SDF adversely affects ART outcomes, with consistent negative associations in IVF and IUI, a weak effect in ICSI, and a strong link to miscarriage in ICSI. These findings clarify the prognostic role of SDF and emphasize the need for standardized assays and large-scale prospective studies before routine clinical implementation.

## Introduction

Infertility affects approximately 10–15% of couples of reproductive age worldwide, with male factors contributing to nearly half of cases [1–3]. Conventional semen parameters—such as sperm concentration, motility, and morphology—remain the primary tools for evaluating male fertility, but they provide only limited insight into the functional competence of spermatozoa [4, 5]. Increasing evidence indicates that sperm DNA integrity is a critical determinant of reproductive success, influencing not only fertilization but also embryo development and the ability to sustain pregnancy [6, 7].

Sperm DNA fragmentation (SDF) has emerged as a widely studied biomarker of sperm nuclear quality. Laboratory assays—including the sperm chromatin structure assay (SCSA), sperm chromatin dispersion (SCD), terminal deoxynucleotidyl transferase dUTP nick end labeling (TUNEL), and the Comet assay—are commonly used to quantify DNA fragmentation. Elevated SDF has been associated with impaired reproductive potential [8, 9]. However, the clinical utility of SDF testing in assisted reproductive technology (ART), including in vitro fertilization (IVF), intracytoplasmic sperm injection (ICSI), and intrauterine insemination (IUI), remains uncertain [10–12].

Over the past decade, multiple meta-analyses have examined the relationship between SDF and ART outcomes. Some reported significant associations between high SDF and reduced clinical pregnancy (CP) or increased miscarriage, whereas others found inconsistent or negligible effects [10, 13–15]. These discrepancies—arising from differences in study design, patient populations, ART procedures, SDF assays, and threshold values—have contributed to heterogeneity and uncertainty in clinical interpretation.

Given the growing number of meta-analyses on this topic, an umbrella review is warranted. Such an approach systematically evaluates and synthesizes the highest level of evidence and can provide a comprehensive overview of the strength, consistency, and credibility of existing findings linking SDF with key ART outcomes, including CP, miscarriage, and live birth rate (LBR).

Therefore, this umbrella meta-analysis aims to the following: (i) summarize and critically appraise published meta-analyses on the impact of SDF on ART outcomes; (ii) evaluate the credibility of evidence across different reproductive endpoints; and (iii) provide clearer guidance for the clinical application of SDF testing in managing infertile couples.

## Materials and methods

### Protocol registration

This umbrella review was conducted in accordance with the Preferred Reporting Items for Systematic Reviews and Meta-Analyses (PRISMA) guidelines and the PRIOR statement for overviews of reviews. The study protocol was prospectively registered in the PROSPERO database (Registration ID: CRD420251142265).

### Literature search

A comprehensive literature search was conducted in PubMed, Embase, Web of Science, Scopus, and the Cochrane Library from inception to July 18, 2025, to identify systematic reviews with meta-analyses evaluating the association between SDF and assisted reproductive outcomes. The search strategy combined Medical Subject Headings and free-text terms, including “sperm DNA fragmentation,” “sperm DNA integrity,” “DNA damage,” “sperm DNA fragmentation index,” “DNA fragmentation index,” “DFI,” “SDF,” “sperm chromatin,” “assisted reproductive technology,” “in vitro fertilization,” “intracytoplasmic sperm injection,” “intrauterine insemination,” “IVF,” “ICSI,” “IUI,” “meta-analysis,” and “systematic review.” Reference lists of eligible studies and related reviews were also manually screened to ensure completeness.

### Eligibility criteria

Two investigators (BW and YF) independently screened titles, abstracts, and full texts, with discrepancies resolved by consensus or consultation with a third reviewer. Eligibility was defined using the PECOS framework: infertile couples undergoing ART procedures (IVF, ICSI, or IUI) (Population); high sperm DNA fragmentation assessed with validated assays such as SCSA, SCD, TUNEL, or Comet (Exposure); low SDF groups (Comparison); CP, pregnancy loss (PL), and LBR (Outcomes); and systematic reviews with meta-analyses of observational or interventional studies (Study design). Exclusion criteria included narrative reviews, systematic reviews without quantitative synthesis, meta-analyses with fewer than two primary studies, studies lacking sufficient data to extract pooled effect estimates [odds ratio (OR), risk ratio (RR), hazard ratio (HR), and 95% confidence interval (CI)], reviews of animal or in vitro studies, and duplicate or overlapping meta-analyses (in which case the most comprehensive or most recent study was retained).

### Data extraction

Two investigators (BW and YF) independently extracted data from all eligible meta-analyses, with disagreements resolved through discussion or adjudication by a third reviewer (Lu WY). Extracted data included the following: first author, year of publication, ART type (IVF, ICSI, or IUI), number of included primary studies and participants, outcomes assessed (CP, PL, or LBR), effect sizes with 95% CIs, measures of heterogeneity (*I*^2^), comparison groups (high vs. low SDF), and details of subgroup or sensitivity analyses.

### Data analysis

All statistical analyses were conducted using the “meta” package in R (version 4.4.2; R Foundation for Statistical Computing, Vienna, Austria). For each eligible meta-analysis, pooled effect estimates were extracted and converted to log risk ratios (logRR) to ensure comparability across studies. Odds ratios and hazard ratios were converted to risk ratios before logarithmic transformation. The corresponding standard errors (SE) and variances (vi) of logRR were calculated from the reported 95% CIs [16]. Heterogeneity was assessed with the *I*^2^ statistic, with *I*^2^ > 50% indicating significant heterogeneity. Summary effect sizes with 95% CIs were calculated using fixed-effect models when *I*^2^ ≤ 50% and random-effects models when *I*^2^ > 50%. A *p*-value < 0.05 was considered statistically significant. In cases of overlapping meta-analyses, robustness of conclusions was assessed through qualitative comparison of findings and certainty ratings across reviews, without additional quantitative synthesis.

### Quality assessment

The methodological quality of included systematic reviews and meta-analyses was assessed using A Measurement Tool to Assess Systematic Reviews 2 (AMSTAR-2), which evaluates 16 domains including protocol registration, comprehensiveness of the literature search, risk of bias assessment, appropriateness of statistical methods, and funding disclosure. Each study was rated as high, moderate, low, or critically low quality [17]. In addition, the certainty of evidence for each ART outcome (CP, PL, and LBR) was evaluated with the Grading of Recommendations, Assessment, Development, and Evaluation (GRADE) framework, which considers risk of bias, inconsistency, indirectness, imprecision, and publication bias. The certainty of evidence was categorized as high, moderate, low, or very low [18]. All evaluations were performed independently by two reviewers, with disagreements resolved by discussion or consultation with a third reviewer. In this umbrella review, our objective was to appraise and contextualize existing meta-analytic evidence rather than to perform de novo quantitative synthesis. Accordingly, we did not re-pool effect estimates across meta-analyses, thereby minimizing the impact of overlapping primary studies and potential double-counting. Robustness of conclusions was assessed qualitatively by comparing the direction and consistency of effects, heterogeneity patterns, and certainty of evidence across reviews.

## Results

### Study selection and characteristics

**Fig. 1:**
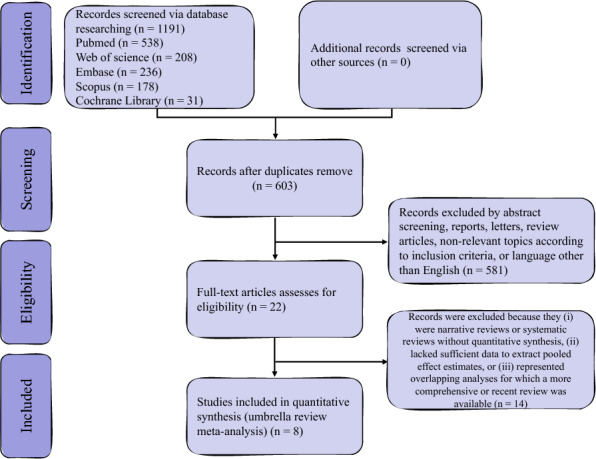
PRISMA flow diagram of study selection. A total of 1191 records were identified across five databases, with 603 remaining after duplicates were removed. After title and abstract screening, 22 full-text articles were reviewed for eligibility, and eight systematic reviews with meta-analyses were ultimately included in this umbrella review

A total of 1191 records were identified through database searches (PubMed *n* = 538; Web of Science *n* = 208; Embase *n* = 236; Scopus *n* = 178; Cochrane Library *n* = 31), with no additional records retrieved from other sources. After duplicates were removed, 603 records remained for title and abstract screening. Of these, 581 were excluded as abstracts, reports, letters, review articles, studies unrelated to SDF or ART, or articles not published in English. The full texts of 22 articles were reviewed, and 14 were excluded because they were narrative reviews, systematic reviews without quantitative synthesis, lacked sufficient data to extract pooled estimates, or represented overlapping analyses. Ultimately, eight systematic reviews with meta-analyses met the inclusion criteria and were included in this umbrella review [7, 10, 11, 14, 15, 19–21]. The study selection process is shown in Fig. 1.

The eight included meta-analyses, published between 2015 and 2025, synthesized data from more than 25,000 ART cycles across IVF, ICSI, and IUI. SDF was assessed using validated assays, including SCSA, SCD, TUNEL, and Comet. The number of primary studies per meta-analysis ranged from 3 to 25, with sample sizes varying from fewer than 500 to more than 7500 cycles. Methodological quality, assessed with AMSTAR-2, ranged from moderate to low, with most reviews rated low due to common shortcomings such as lack of protocol registration, incomplete literature searches, and insufficient assessment of publication bias. Certainty of evidence, appraised with the GRADE framework, was uniformly rated as low. This downgrading was mainly due to the predominance of retrospective, non-randomized studies, together with substantial heterogeneity, imprecision, inconsistencies across ART subgroups, and other methodological limitations. Collectively, these findings suggest that although elevated SDF is associated with adverse ART outcomes, the overall credibility of the evidence remains limited (Table 1).

**Table 1:** Characteristics of included meta-analyses assessing sperm DNA fragmentation and ART outcomes

First author	Year	ART type	Outcome	No. of studies in MA	Measure	Effect size (95% CI)	Sample size	*p* value	*I*^2^	Comparison	AMSTAR-2	GRADE
Zhang et al. [14]	2015	IVF	CP	9	OR	1.742 (1.382–2.195)	2751	0.000	56.1%	Low vs. high	Moderate	Low
		ICSI	CP	5	OR	0.895 (0.629–1.273)	765	0.537	0%	Low vs. high		
		IVF	PL	4	OR	1.262 (0.58–2.704)	811	0.549	0%	Low vs. high		
		ICSI	PL	4	OR	0.542 (0.290–1.013)	501	0.055	0%	Low vs. high		
Deng et al. [7]	2019	IVF	LBR	5	RR	0.84 (0.67–1.06)	1823	0.15	63%	High vs. low	Moderate	Low
		IVF	PL	7	RR	1.68 (1.19–2.36)	1779	0.003	35%	High vs. low		
		ICSI	PL	4	RR	3.38 (1.45–7.85)	113	0.005	0%	High vs. low		
Ribas-Maynou et al. [11]	2021	IVF	CP	15	RR	0.72 (0.55–0.95)	5255	0.02	72%	High vs. low	Low	Low
		ICSI	CP	25	RR	0.89 (0.78–1.02)	7590	0.09	44%	High vs. low		
		IVF	LBR	6	RR	0.48 (0.22–1.02)	2062	0.06	79%	High vs. low		
		ICSI	LBR	9	RR	0.92 (0.67–1.27)	4062	0.62	70%	High vs. low		
Chen et al.	2022	IVF	CP	7	RR	0.79 (0.67–0.93)	2936	0.005	73%	High vs. low	Moderate	Low
		ICSI	CP	10	RR	0.95 (0.74–1.24)	3630	0.72	45%	High vs. low		
		IVF	LBR	3	RR	0.53 (0.16–1.80)	932	0.31	76%	High vs. low		
		ICSI	LBR	3	RR	0.99 (0.61–1.60)	2895	0.97	73%	High vs. low		
Simon et al.	2017	IVF	CP	16	RR	1.92 (1.33–2.77)	3734	0.0005	60.7%	Low vs. high	Low	Low
		ICSI	CP	25	RR	1.49 (1.11–2.01)	2282	0.0075	48.7%	Low vs. high		
Chen et al. [20]	2019	IUI	CP	10	RR	0.34 (0.22–0.52)	2839	0.000	1.2%	High vs. low	Low	Low
Liu et al. [10]	2025	IUI	CP	7	RR	0.82 (0.52–1.29)	4541	0.32	41%	High vs. low	Moderate	Low
			PL	3	RR	2.11 (0.93–4.80)	399	0.07	0%	High vs. low		
Sugihara et al. [19]	2020	IUI	CP	3	RR	3.30 (1.16–9.39)	917	NR	38%	Low vs. high	Low	Low

*ART* assisted reproductive technology, *IVF* in vitro fertilization, *ICSI* intracytoplasmic sperm injection, *IUI* intrauterine insemination, *CP* clinical pregnancy, *PL* pregnancy loss, *LBR* live birth rate, *OR* odds ratio, *RR* risk ratio, *CI* confidence interval, *NR* not reported, *MA* meta-analysis, *AMSTAR-2* Assessment of Multiple Systematic Reviews, version 2, *GRADE* Grading of Recommendations Assessment, Development, and Evaluation

### Association between SDF and CP

**Fig. 2:**
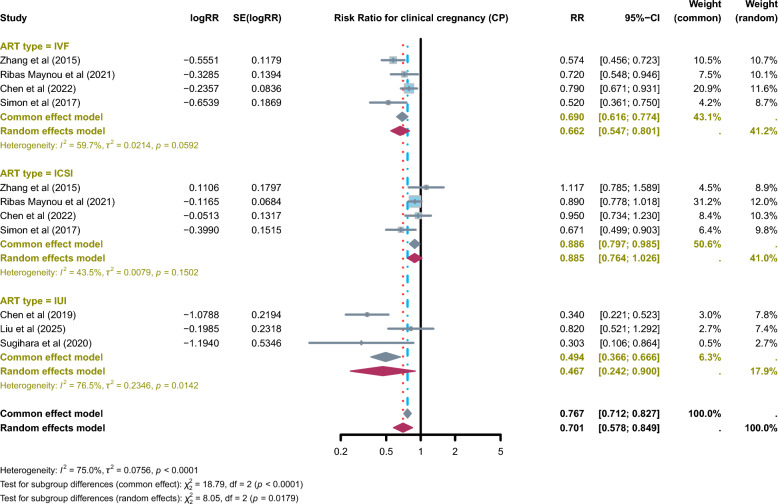
Reported pooled effect estimates from published meta-analyses evaluating the association between sperm DNA fragmentation (SDF) and clinical pregnancy (CP) across assisted reproductive technology (ART) modalities. This figure provides a descriptive visual summary of pooled risk ratios (RRs) as reported in individual meta-analyses, stratified by IVF, ICSI, and IUI. No de novo quantitative synthesis, re-weighting, or secondary meta-analysis was performed at the umbrella review level. The figure is intended to facilitate qualitative comparison of effect direction, magnitude, and heterogeneity across reviews rather than to generate an overall pooled estimate

Seven meta-analyses examined the association between SDF and CP across ART procedures. The included meta-analyses showed that elevated SDF was significantly associated with a lower likelihood of achieving CP (RR = 0.701, 95% CI: 0.578–0.849; *I*^2^ = 75.0%), although heterogeneity was substantial. Subgroup analyses revealed distinct patterns. In IVF cycles, high SDF was consistently associated with significantly reduced CP rates (RR = 0.662, 95% CI: 0.547–0.801; *I*^2^ = 59.7%). In ICSI cycles, a weak, borderline association was observed (RR = 0.886, 95% CI: 0.764–0.985; *I*^2^ = 43.5%). In IUI, elevated SDF was also significantly associated with reduced CP rates (RR = 0.467, 95% CI: 0.242–0.900; *I*^2^ = 76.5%), though the high heterogeneity indicates considerable uncertainty. Subgroup analyses by ART modality (IVF, ICSI, and IUI) were consistently reported across included meta-analyses; however, subgroup or meta-regression analyses stratified by SDF assay type were inconsistently performed or not uniformly available, precluding further quantitative exploration at the umbrella review level. Overall, these findings suggest that SDF negatively affects CP in IVF and IUI, while in ICSI only a weak, borderline association was observed (Fig. 2).

### Association between SDF and PL

**Fig. 3:**
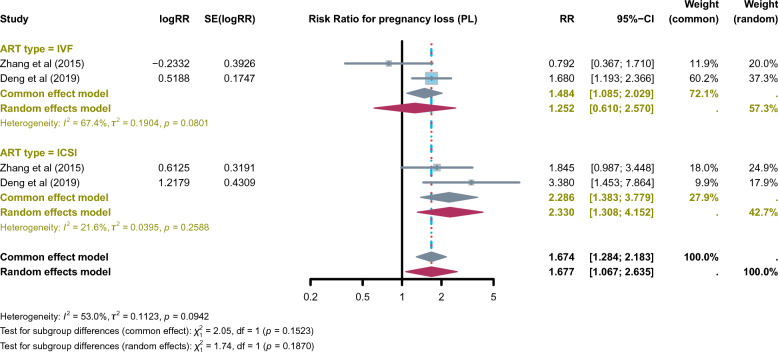
Reported pooled effect estimates from published meta-analyses assessing the association between sperm DNA fragmentation (SDF) and pregnancy loss (PL) in assisted reproductive technology (ART). Effect estimates shown are extracted directly from individual meta-analyses and displayed descriptively by ART modality. This figure does not represent a secondary quantitative re-analysis or umbrella-level pooling, but serves as a qualitative evidence summary to compare consistency and direction of associations across reviews

Two meta-analyses assessed the association between SDF and PL in ART cycles (IVF and ICSI). The included meta-analyses showed a significant increase in miscarriage risk among couples with elevated SDF (RR = 1.677, 95% CI: 1.067–2.635; *I*^2^ = 53%) (Fig. 3). When stratified by ART type, no significant association was found in IVF cycles (RR = 1.252, 95% CI: 0.610–2.570; *I*^2^ = 67.4%). In contrast, in ICSI cycles, high SDF was significantly associated with increased PL risk (RR = 2.286, 95% CI: 1.383–3.779; *I*^2^ = 21.6%). These findings suggest a consistent adverse impact of SDF on miscarriage, particularly in ICSI, where natural sperm selection is bypassed [22].

### Association between SDF and LBR

**Fig. 4:**
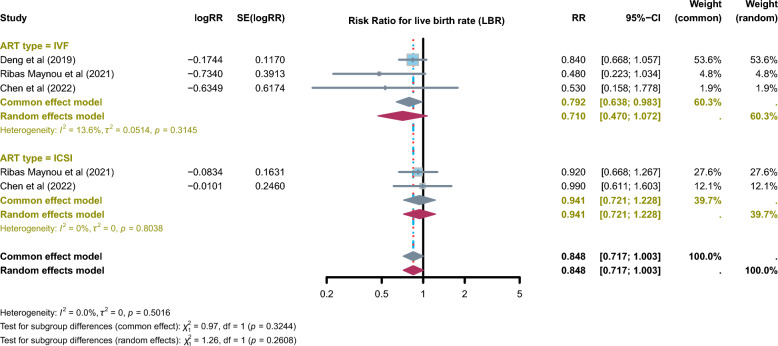
Descriptive summary of pooled effect estimates reported by individual meta-analyses on the association between sperm DNA fragmentation (SDF) and live birth rate (LBR) in assisted reproductive technology (ART). All risk ratios (RRs) are presented as reported in the original meta-analyses. No additional statistical pooling or recalculation was conducted for this umbrella review. The figure is used solely to support qualitative interpretation alongside AMSTAR-2 and GRADE assessments of evidence certainty

Three meta-analyses examined the association between SDF and LBR in ART cycles (IVF and ICSI). The overall pooled estimate indicated a non-significant trend toward reduced LBR in couples with high SDF (RR = 0.848, 95% CI: 0.717–1.003; *I*^2^ = 0%). Subgroup analyses showed that in IVF cycles, elevated SDF was significantly associated with a lower likelihood of live birth (RR = 0.792, 95% CI: 0.638–0.983; *I*^2^ = 13.6%), whereas in ICSI cycles, no significant association was observed (RR = 0.941, 95% CI: 0.721–1.228; *I*^2^ = 0%). These results are shown in Fig. 4.

### Evidence certainty

According to the GRADE framework, the certainty of evidence across all outcomes was uniformly rated as low. For CP, pooled analyses indicated significant reductions in IVF and IUI, and only a weak, borderline association in ICSI. The evidence was downgraded due to inconsistency across ART subgroups, substantial heterogeneity, and imprecision. For PL, the overall effect suggested an increased risk with elevated SDF, particularly in ICSI cycles; however, certainty was rated as low given the limited number of meta-analyses, variability between IVF and ICSI findings, and methodological shortcomings of the underlying studies. For LBR, the evidence was also judged as low certainty, as three meta-analyses reported this outcome, all with relatively small sample sizes, wide CIs, and no data available for IUI. Taken together, these findings indicate that although high SDF is suggestively associated with adverse ART outcomes, the overall strength and reliability of the evidence remain limited.

## Discussion

This umbrella meta-analysis provides a comprehensive synthesis of the evidence on the association between SDF and key ART outcomes, namely CP, PL, and live birth. By systematically evaluating eight published meta-analyses, we generated pooled estimates across IVF, ICSI, and IUI and critically appraised the credibility of these findings using the AMSTAR-2 and GRADE frameworks.

Our findings indicate that elevated SDF is significantly associated with reduced CP rates in IVF and IUI, with only a weak, borderline association in ICSI. These results are consistent with earlier systematic reviews reporting adverse effects of SDF on CP in conventional insemination, but less consistent evidence for ICSI [7, 19, 20]. The pooled analysis also showed that high SDF is associated with an increased risk of PL in ART, with the strongest effect in ICSI, consistent with recent evidence that elevated SDF increases miscarriage risk following ICSI [7, 23]. For live birth, the evidence was less consistent: Although IVF cycles showed a significant reduction in LBR with high SDF, the overall pooled estimates did not reach statistical significance, and CIs frequently crossed unity. Notably, no meta-analysis specifically evaluated live birth in IUI, highlighting a critical gap in the literature.

Comparison with previous meta-analyses shows broad agreement that SDF negatively influences ART outcomes, particularly CP and PL [7, 24, 25]. However, the magnitude and consistency of these associations vary across ART modalities. In IVF, the evidence for reduced CP and lower LBR aligns with prior reports, supporting SDF as a potential prognostic marker in this setting [26–29]. In ICSI, although earlier reviews reported conflicting results [15], our umbrella review suggests that SDF shows only a weak, borderline association with CP, while being more strongly linked to PL than to reduced CP, highlighting the complexity of interpreting SDF in the context of sperm selection and micromanipulation [23, 30, 31]. In IUI, despite fewer studies, the association between high SDF and reduced CP [19, 20] is consistent with biological expectations given the reliance on natural sperm selection. This pattern may reflect fundamental biological differences among ART modalities. In IVF and IUI, fertilization depends more on natural sperm selection mechanisms, whereby sperm with extensive DNA damage are less likely to achieve successful fertilization, leading primarily to reduced CP. In contrast, ICSI bypasses physiological sperm selection, potentially attenuating the impact of SDF on fertilization while allowing unrepaired paternal DNA damage to manifest at later developmental stages, thereby increasing the risk of PL.

Despite these associations, the certainty of evidence across outcomes was predominantly graded as low by the GRADE framework. This downgrading was due to several factors. First, most primary studies included in the meta-analyses were observational, often retrospective, and therefore prone to bias. Second, substantial heterogeneity was observed, particularly for CP, reflecting differences in patient populations, laboratory methods, SDF thresholds, and ART protocols. Although subgroup analyses by ART type were feasible and clinically informative, further stratification by SDF assay type (e.g., SCSA, TUNEL, Comet) was limited by inconsistent reporting and heterogeneous cut-off definitions across primary studies and meta-analyses. Consequently, assay-specific effects were addressed qualitatively rather than through additional quantitative subgroup analyses. Third, the precision of pooled estimates was limited by small sample sizes, especially for LBR, which was reported in only three reviews. Fourth, methodological shortcomings of the included reviews, reflected by predominantly low AMSTAR-2 ratings, further reduced confidence. Taken together, these limitations suggest that although high SDF is repeatedly associated with adverse ART outcomes, the robustness of the evidence remains limited. In view of the low certainty of evidence, SDF testing should be considered an adjunctive, context-dependent tool rather than a routine or stand-alone test, and its results should primarily support counseling rather than dictate clinical management.

From a clinical perspective, these findings have several implications. SDF testing may serve as a complementary biomarker in the evaluation of male infertility, particularly in settings such as unexplained infertility, recurrent implantation failure, or recurrent PL [31–34]. The stronger associations observed in IVF and IUI compared with ICSI suggest that the clinical utility of SDF testing is context-dependent and should be interpreted with caution in ICSI populations. Furthermore, the weak and inconsistent evidence for live birth highlights the need for future studies to move beyond intermediate outcomes and prioritize clinically meaningful endpoints. However, current clinical guidelines adopt a cautious and targeted approach to SDF testing. Routine SDF testing is generally discouraged during the initial infertility evaluation, as evidence that testing alone improves clinical outcomes remains limited. By contrast, guidelines support considering SDF testing in selected scenarios, such as recurrent pregnancy loss, unexplained infertility, or repeated failure of assisted reproductive technologies, where SDF may provide additional information beyond conventional semen parameters. Within this context, our findings support a scenario-based rather than universal application of SDF testing, pending high-quality prospective evidence demonstrating that SDF-guided interventions improve live birth outcomes.

Several limitations of this umbrella review should be noted. First, our analysis relied on previously published meta-analyses, so the strength of our conclusions depends on the methodological rigor of those reviews. Second, substantial heterogeneity was evident across studies, stemming from differences in study populations, semen parameters, female partner characteristics, laboratory methods, ART protocols, and follow-up durations. An important source of heterogeneity was the lack of standardized thresholds for defining high versus low SDF, with individual studies applying different cut-off values that limited comparability. Third, multiple assays were used to assess SDF (SCSA, SCD, TUNEL, Comet), each with unique sensitivity, specificity, and technical limitations, complicating cross-study synthesis. Fourth, most primary studies were observational, often retrospective, and therefore vulnerable to selection bias, residual confounding, and unmeasured variables. Finally, although methodological quality was assessed using AMSTAR-2 and GRADE, many reviews were rated as low, reflecting recurring shortcomings such as lack of preregistered protocols, incomplete literature searches, limited bias assessment, and insufficient evaluation of publication bias. Collectively, these limitations highlight the urgent need for well-designed, prospectively registered, and methodologically rigorous studies to produce more robust evidence.

In addition, several methodological considerations specific to umbrella reviews merit attention. Overlap of primary studies across meta-analyses is unavoidable; however, because no secondary quantitative pooling was performed in the present study, its influence on effect estimation is limited. Nevertheless, overlap and heterogeneity together underscore the need for standardized SDF testing, harmonized cut-off values, and prospectively registered, methodologically rigorous studies to generate more robust and clinically actionable evidence.

Future research should address these limitations by conducting large-scale, prospective, and ideally randomized studies using standardized SDF assays and clearly defined thresholds. Harmonization of outcome reporting, particularly for live birth, is urgently needed, along with investigations into the modifying effects of female factors, embryo selection strategies, and ART protocols. Collaborative registries and multicenter trials will be essential for generating robust and generalizable evidence to guide clinical practice.

In summary, this umbrella meta-analysis indicates that elevated sperm DNA fragmentation is associated with reduced clinical pregnancy in IVF and IUI, shows a weak, borderline association with clinical pregnancy in ICSI, and is strongly linked with increased miscarriage risk, particularly in ICSI. Evidence for live birth remains limited and inconclusive. Although these findings support a potential role for SDF as a prognostic marker in ART, the certainty of evidence is low, underscoring the need for standardized assays and well-designed prospective studies before routine clinical implementation.

## Data Availability

No datasets were generated or analysed during the current study.
